# α‐synuclein pathology drives region‐specific tau deposition

**DOI:** 10.1002/alz.71932

**Published:** 2026-10-25

**Authors:** Seyed Hani Hojjati, Gloria C. Chiang, Xiuyuan Hugh Wang, Mohammad Khalafi, Sudhin Shah, Liangdong Zhou, Samantha Keil, Lidia Glodzik, Laura Beth J. McIntire, Emily Tanzi, Farnia Feiz, Tom Maloney, Yi Li, Tracy A. Butler, for the Alzheimer's Disease Neuroimaging Initiative

**Affiliations:** ^1^ Department of Radiology Brain Health Imaging Institute Weill Cornell Medicine New York New York USA

**Keywords:** α‐synuclein, Alzheimer's disease, dementia with Lewy bodies, seed amplification assay, tau positron emission tomography

## Abstract

**INTRODUCTION:**

Dementia with Lewy bodies (DLB) is a common but underdiagnosed neurodegenerative disease associated with α‐synuclein pathology. Clinically, DLB is characterized by early motor dysfunction and psychiatric symptoms, in contrast to early memory decline typical of Alzheimer's disease (AD). Lewy body and AD pathology commonly co‐occur. It remains unclear how α‐synuclein pathology, either alone or in combination with AD pathology, produces clinical features considered distinctive of synucleinopathy.

**METHODS:**

We analyzed 531 Alzheimer's Disease Neuroimaging Initiative participants using neuroimaging to compare tau deposition patterns between α‐synuclein–positive and α‐synuclein–negative individuals.

**RESULTS:**

α‐synuclein–positive individuals showed significantly greater tau deposition in basal ganglia (BG), insula, and medial temporal lobe (MTL). Increased tau burden in these regions correlated with more severe psychiatric symptoms and slower reaction times. These associations were present only in the α‐synuclein–positive group.

**DISCUSSION:**

These results suggest that α‐synuclein pathology is associated with region‐specific tau positron emission tomography abnormalities that map onto core clinical features of synucleinopathy.

## Highlights

- The presence of α‐synuclein co‐pathology is linked to significantly elevated tau deposition in the basal ganglia, insula, and medial temporal lobe.
- Findings provide human in vivo evidence supporting preclinical models in which α‐synuclein promotes the cross‐seeding and anatomical spread of tau aggregates.
- Higher regional tau burden in α‐synuclein–positive individuals is uniquely associated with increased psychiatric symptoms and slower psychomotor task reaction times.
- Neuroimaging‐based topographical signatures of tau can serve as a proxy for identifying α‐synuclein co‐pathology during life.

## 1 BACKGROUND

Lewy bodies composed of α‐synuclein are the pathological hallmark of neurodegenerative synucleinopathies, the two most common of which are Parkinson's disease (PD) and dementia with Lewy bodies (DLB).^1^, ^2^ PD is characterized by progressive motor dysfunction, including slowing of movement, tremor, and loss of balance. In DLB, motor dysfunction can be subtle initially, while neuropsychiatric symptoms including depression and psychosis can be prominent. While accurate diagnosis of PD is typically possible based on distinctive clinical features, DLB remains substantially under‐recognized in clinical practice.^3^ Previous reports have indicated that up to one third of DLB cases are initially missed, with many patients receiving alternative diagnoses before α‐synuclein–related disease is identified.^4^ At autopsy, α‐synuclein pathology, either in isolation or as a co‐pathology, is highly prevalent in patients diagnosed with Alzheimer's disease (AD) or other neurodegenerative diseases, and even in healthy older adults. Unlike AD, there are no positron emission tomography (PET) diagnostic tests for synucleinopathies. Early‐stage experimental tracers for α‐synuclein, such as [^18^F] ACI‐12589, are not yet established for clinical or research use.^5^, ^6^ Conventional biomarkers for PD and DLB, including dopamine transporter (DAT) neuroimaging and cardiac 123I‐MIBG (metaiodobenzylguanidine) scintigraphy, primarily capture downstream effects of α‐synuclein rather than α‐synuclein pathology itself.^7^ Recently, accurate and specific in vivo biomarker tests for synucleinopathy have become available.

Synucleinopathy can now be diagnosed using seed amplification assay (SAA) performed on cerebrospinal fluid (CSF) obtained via lumbar puncture (LP)^8^ as well as by skin biopsy.^9^, ^10^ SAA has been shown to accurately detect α‐synuclein seeding activity across the spectrum of synucleinopathies including DLB, PD, isolated rapid eye movement sleep behavior disorder (iRBD), and pure autonomic failure (PAF), with an overall sensitivity of 95.3%. The SAA assay demonstrated 98% specificity in a neuropathological cohort of 101 cases lacking synuclein pathology.^8^, ^11^ Beyond the binary determination of synucleinopathy presence versus absence using SAA, CSF DOPA‐decarboxylase (DDC) has recently emerged as a quantitative fluid biomarker reflecting α‐synuclein disease. A recent study found that the CSF concentration of DDC measured using two immunoassays was strongly associated with α‐synuclein burden at autopsy, particularly in the substantia nigra, and distinguished DLB and PD from both controls and AD patients with area under the curve > 0.9.^12^ Because CSF DDC may reflect the functional consequences of α‐synuclein pathology on dopaminergic systems rather than directly detecting misfolded α‐synuclein, CSF DDC and SAA may serve as complementary biomarkers. These tests are not yet in widespread clinical use, and some patients consider them invasive. Consequently, the majority of individuals with α‐synuclein pathology remain undiagnosed, and the contribution of α‐synuclein pathology to in vivo measurements of brain structure and function remains uncertain.

Because there are only a few in vivo neuroimaging studies of individuals with confirmed α‐synuclein pathology, understanding the neural basis of symptomatology and interactions between α‐synuclein and other neurodegenerative pathologies is extremely limited. Emerging evidence indicates that α‐synuclein co‐pathology is associated with greater amyloid beta (Aβ) deposition^13^ and Aβ‐driven tau deposition,^14^ exacerbates glucose hypometabolism,^15^ and drives faster clinical decline.^16^ Collectively, these studies highlight a potential pathogenic interplay among α‐synuclein, tau, and Aβ in driving heterogeneous clinical phenotypes and shared mechanisms of neurodegeneration across multiple dementia syndromes and are in accord with experimental studies showing that α‐synuclein induces tau hyperphosphorylation and accelerates tau propagation.^17^, ^18^

However, prior studies have generally focused on broad categories of individuals categorized as tau or Aβ positive or negative, whereas it is well established that regional distribution of tau is critically important to the nature and severity of neuropsychiatric deficits in AD.^19^, ^20^ It is largely unexamined whether the presence of α‐synuclein pathology affects regional patterns of tau deposition across the whole brain using voxel‐wise analyses, and how such patterns relate to clinical manifestations, including motor and psychiatric features known to be common in synucleinopathies.^1^, ^2^, ^7^ We hypothesize that α‐synuclein pathology, together with its synergistic interactions with AD pathology, drives region‐specific alterations in tau aggregation. Using voxel‐wise analyses of AV‐1451 (tau) PET, we map how α‐synuclein associates with tau deposition across the whole brain and assess the clinical relevance of PET patterns by correlating them with two key clinical features of synucleinopathy: motor slowing and psychiatric symptoms.

## 2 METHODS

### 2.1 Participants

**RESEARCH IN CONTEXT**

1. **Systematic review**: Current evidence from neuropathological and biochemical studies confirms that the co‐occurrence of α‐synuclein and tau pathologies is a hallmark of neurodegenerative aging, frequently co‐existing in patients diagnosed clinically with Alzheimer's disease (AD). While preclinical models have established that α‐synuclein can promote the cross‐seeding and accelerated propagation of tau aggregates, translating these findings to living human cohorts has proven challenging. To date, human in vivo research has largely focused on single‐protein models, leaving a critical gap in understanding the specific whole‐brain anatomical hubs where α‐synuclein modifies tau topography. Furthermore, the degree to which this protein interaction drives specific clinical symptoms beyond standard memory loss, such as psychomotor and psychiatric disturbances, remains insufficiently characterized in large‐scale clinical cohorts.
2. **Interpretation**: This study identifies a localized topographical signature of α‐synuclein–associated tau accumulation within the basal ganglia, insula, and medial temporal lobe. The findings suggest that the presence of α‐synuclein does not merely increase global tau burden but specifically targets regions linked to psychomotor speed and psychiatric regulation. By linking these regional tau elevations to more severe psychiatric symptoms and slower reaction times, this research provides a mechanistic basis for the clinical heterogeneity observed in mixed‐pathology dementias, distinguishing them from pure AD phenotypes.
3. **Future directions**: The results emphasize the necessity of neuroimaging‐based phenotyping for the in vivo detection of mixed proteinopathies. Future longitudinal studies should investigate whether these α‐synuclein–related tau signatures can serve as predictive biomarkers for disease progression. Additionally, these findings advocate for the inclusion of multi‐modal imaging in clinical trials to ensure that therapeutic interventions are tailored to the specific pathological profile of the individual, particularly when α‐synuclein–targeted therapies are considered.

In this study, we included 531 participants (mean age 73.72 ± 7.6 years; 277 females) from the Alzheimer's Disease Neuroimaging Initiative (ADNI; adni.loni.usc.edu) who had undergone CSF α‐synuclein assay as well as tau PET (mean time difference from the CSF exam date: 1.48 ± 2.31 years), Aβ PET (mean time difference from the CSF exam date: 0.23 ± 0.7), and structural magnetic Resonance Imaging (MRI). ADNI, launched in 2003 as a public–private partnership led by Principal Investigator Michael W. Weiner, MD, aims to determine whether serial MRI, PET, other biological markers, and clinical and neuropsychological assessment can be combined to measure the progression of mild cognitive impairment (MCI) and early AD. The current goals include validating biomarkers for clinical trials, improving the generalizability of ADNI data by increasing diversity in the participant cohort, and providing data concerning the diagnosis and progression of AD to the scientific community. The cohort for this analysis consisted of 293 cognitively healthy controls (HC), 180 individuals with MCI, and 58 with AD; 102 were α‐synuclein positive and 429 were α‐synuclein negative; 236 were Aβ positive, and 295 were Aβ negative.^12^ Participants were classified as HC (Clinical Dementia Rating [CDR] = 0, Mini‐Mental State Examination [MMSE] 24–30, with no significant subjective or objective cognitive or functional impairment), MCI (CDR = 0.5 with a memory box score ≥ 0.5, MMSE 24–30, objective memory impairment on education‐adjusted delayed recall, and preserved general cognition and functional independence, per Petersen/ADNI criteria), or AD dementia (CDR 0.5 or 1.0, MMSE 20–26, meeting National Institute of Neurological and Communicative Disorders and Stroke–Alzheimer's Disease and Related Disorders Association criteria for probable AD). Aβ positivity was determined using Aβ status information from University of California Berkeley–processed Aβ PET data in the ADNI dataset.

### 2.2 Participant assessments

For this study, we focused on two key clinical domains strongly implicated in synucleinopathy: psychiatric symptoms and motor functioning.^1^, ^7^ Psychiatric symptoms were assessed using the Neuropsychiatric Inventory (NPI) total score. Although sensitive measures of motor function are not included in the ADNI assessment battery, and individuals with overt parkinsonism at baseline are excluded from participation as ADNI is focused on AD, we estimated psychomotor speed as an available proxy for motor function based on three tests in the CogState^21^ computerized battery (detection, identification, and one‐back) that measured reaction time.^21^ While reaction time on these tasks reflects a combination of processing speed, attention, response selection, and motor output rather than motor function in isolation,^22^ reaction time is well established to be prolonged in the most common synucleinopathy, PD, though less markedly impaired than movement time.^23^ Further supporting the use of reaction time as an available measure of motor/psychomotor functioning when dedicated motor measures are unavailable, reaction time on the Cogstate detection task has been strongly associated with grooved pegboard performance (*r* = 0.81), a well‐established test of manual dexterity and fine motor function.^22^ Principal component analysis (PCA) was performed to capture shared variance in reaction time across the three tasks and define a robust composite measure of psychomotor speed.^22^

### 2.3 α‐synuclein status

α‐synuclein positivity was determined using the categorical SAA result distributed by ADNI, generated with the Amprion CSF‐based seed amplification assay,^11^ which detects misfolded α‐synuclein seeds through cyclic amplification of a recombinant α‐synuclein substrate. α‐synuclein pathology aggregation was coded as a binary status (α‐synuclein positive and α‐synuclein negative).

### 2.4 Clinical comparisons between α‐synuclein–positive and α‐synuclein–negative groups

We compared demographic (e.g., age, sex), cognitive (MMSE), psychiatric (NPI total score), and psychomotor speed (composite reaction time) features between α‐synuclein–positive and α‐synuclein–negative individuals. The Shapiro–Wilk test was used to assess the normality of continuous measures, for which a significance threshold of *p* < 0.05 indicated a violation of normality assumptions. Accordingly, group differences were evaluated using independent *t* tests for normally distributed data and the Mann–Whitney *U* test for non‐normally distributed variables. Categorical measures were analyzed using the chi‐squared test.

### 2.5 Tau PET processing and analysis

AV‐1451 PET scans were acquired beginning 75 minutes post‐injection and included six 5‐minute frames. PET images were processed using a customized PETSurfer ^24^ pipeline incorporating FSL,^25^ FreeSurfer, and Advanced Normalization Tools (ANTs). ^26^ First, individual frames were aligned to the first frame and summed to create a single PET volume. The summed PET images were rescaled using the cerebellar gray matter as the reference region. PET images were transformed into MNI152 1 mm space for standardized spatial analyses using ANTs.

Voxel‐wise analyses were conducted using the generalized linear model (GLM) in Statistical Parametric Mapping (SPM).^27^ α‐synuclein–positive and α‐synuclein–negative participants were compared, adjusting for age, sex, and Aβ status as covariates. To assess the potential impact of the time difference between CSF and tau PET, we also repeated the analyses including the time interval between the CSF exam and tau PET acquisition dates as a covariate. Clusters were performed using a voxel‐level threshold of p < 0.001 (uncorrected), and cluster‐level inference was corrected for multiple comparisons using the false discovery rate (FDR), with significance set at FDR‐corrected p < 0.05.  Finally, as a supplementary analysis accounting for AD co‐pathologies, we repeated the analyses separately in the Aβ‐positive and Aβ‐negative groups.

### 2.6 Correlation between tau PET signal and clinical features

To clarify the clinical correlations of regional differences in tau PET identified through voxel‐wise analysis, we examined the association between clinical features (psychiatric symptoms and psychomotor speed) and tau PET signal in anatomically constrained clusters identified in voxel‐wise comparisons. After assessing distributional normality using the Shapiro–Wilk test to determine the appropriate correlation method (Pearson or Spearman), partial correlation analyses controlling for age, sex, and Aβ status were used to assess the association of psychiatric symptom severity (NPI total score) and psychomotor speed with regional tau PET signal. Psychomotor speed was indexed by the first principal component (PC1) derived from reaction times on the three CogState tasks (detection, identification, and one‐back), with the proportion of variance explained and task loadings reported in the Results section. These analyses were conducted for all participants, as well as separately for α‐synuclein–negative and α‐synuclein–positive groups. The Benjamini–Hochberg procedure was applied to control the FDR.

### 2.7 Sensitivity analyses

To characterize the magnitude of each association, partial Spearman correlations were converted to an equivalent Cohen *d*, and effect sizes were compared between the α‐synuclein–positive and α‐synuclein–negative groups.

To address whether tau PET signal varies across the AD continuum among biologically defined (Aβ‐positive) participants, we used the non‐parametric Kruskal–Wallis test to assess the overall difference in tau PET signal across the three clinical stages (HC, MCI, AD) within Aβ‐positive participants.

## 3 RESULTS

### 3.1 Participant demographics, clinical characteristics, and comparisons between α‐synuclein–positive and α‐synuclein–negative groups

Table 1 summarizes the demographic and clinical characteristics of the study participants and group differences based on α‐synuclein status. Of 531 participants, 400 participants (333 α‐synuclein negative and 67 α‐synuclein positive) completed clinical and cognitive assessments. The three CogState reaction time tasks impose relatively low cognitive load but still reflect a combination of motor execution and cognitive processing. Reaction times across the three tasks were moderately correlated (*r* > 0.51, *p* < 0.0001), indicating substantial shared variance while leaving room for task‐specific cognitive demands. To isolate the shared motor component from this task‐specific variance, we performed principal component analysis (PCA) on the reaction times and examined all three components (Table 2). PC1 accounted for 74% of the total variance and showed positive loadings across all three tasks (detection = 0.56, identification = 0.60, one‐back = 0.56), supporting its interpretation as a shared psychomotor component, with lower PC1 scores corresponding to faster reaction times. In contrast, PC2 and PC3 showed mixed positive and negative loadings, consistent with task‐specific cognitive demands. PC1 was therefore retained as the psychomotor speed index in subsequent analyses.

**TABLE 1:** Participants’ clinical characteristics.

​​	α‐synuclein negative	α‐synuclein positive	*p* value
Number of individuals	333	67	—
Age (mean ± SD)	72.82 ± 7.45	75.10 ± 6.41	*p* = 0.014^a^
Sex (male/female)	144 / 189	37 / 30	*p* = 0.1^b^
Number of HC/MCI/AD	213 / 111 / 9	44 / 21 / 2	*p* = 0.95^b^
Aβ positive within HC, *n*/ total *N* (%)	62/213 (29.0%)	15/44 (34.1%)	*p* = 0.59^b^
Aβ positive within MCI, *n*/ total *N* (%)	53/111 (48%)	11/21 (52.4%)	*p* = 0.81^b^
Aβ positive within AD, *n*/ total *N* (%)	7/9 (77.8%)	2/2 (100.0%)	*p* = 1.00^b^
MMSE score (mean ± SD)	28.57 ± 2.13	28.40 ± 2.50	*p* = 0.84 ^a^
NPI total score (mean ± SD)	2.36 ± 5.63	2.96 ± 4.61	*p* = 0.08 ^a^
Response time PC1 (mean ± SD)	0 ± 0.17	0.02 ± 0.16	*p* = 0.39 ^a^
Detection response time (mean ± SD)^c^	2.62 ± 0.12	2.63 ± 0.11	*p* = 0.42 ^a^
Identification response time (mean ± SD)^c^	2.78 ± 0.08	2.78 ± 0.07	*p* = 0.77 ^a^
One back response time (mean ± SD)^c^	2.95 ± 0.10	2.97 ± 0.11	*p* = 0.30 ^a^

Abbreviations: Aβ, amyloid beta; AD, Alzheimer's disease; HC, healthy control; MCI, mild cognitive impairment; MMSE, Mini‐Mental State Examination; NPI, Neuropsychiatric Inventory; PC1, first principal component; SD, standard deviation.

^a^ Mann–Whitney *U* test

^b^Chi‐squared test

**^c^** Unit: log_10_(milliseconds).

**TABLE 2:** Task loadings in principal component analysis.

​​ Tasks	PC1 load	PC2 load	PC3 load
Detection reaction time	0.56	0.71	0.42
Identification reaction time	0.60	−0.005	−0.80
One‐back reaction time	0.56	−0.70	0.43

Abbreviation: PC, principal component.

All continuous demographic and clinical measures and regional tau depositions showed significant deviation from normality (*p* < 0.02); therefore, we used non‐parametric tests for all analyses.

The α‐synuclein–positive group was significantly older than the α‐synuclein–negative group (75.10 ± 6.41 versus 72.82 ± 7.45 years; *p* = 0.014), while sex distribution, clinical diagnosis, and Aβ status did not differ between groups (*p* ≥ 0.1).

There was a trend toward more severe psychiatric symptoms (NPI total score) in the α‐synuclein–positive group with marginal significance (2.96 ± 4.61 vs. 2.36 ± 5.63; *p* = 0.08). No significant group differences were observed in PC1 or MMSE scores.

### 3.2 Voxel‐wise analyses comparing tau PET in α‐synuclein–positive versus α‐synuclein–negative groups

To provide a visual complement to the statistical analyses, we generated group‐average tau PET standardized uptake value ratio (SUVR) maps for α‐synuclein–positive (Figure 1A, *n* = 102) and α‐synuclein–negative (Figure 1B, *n* = 429) participants. Both groups showed the expected overall distribution of tau PET signal, with the highest SUVR values concentrated in temporal and subcortical gray matter. Visual inspection of the difference (Figure 1C) map (α‐synuclein positive—α‐synuclein negative) showed elevated signal in α‐synuclein–positive participants concentrated in the basal ganglia (BG), insula, and medial temporal lobe (MTL).

**FIGURE 1:**
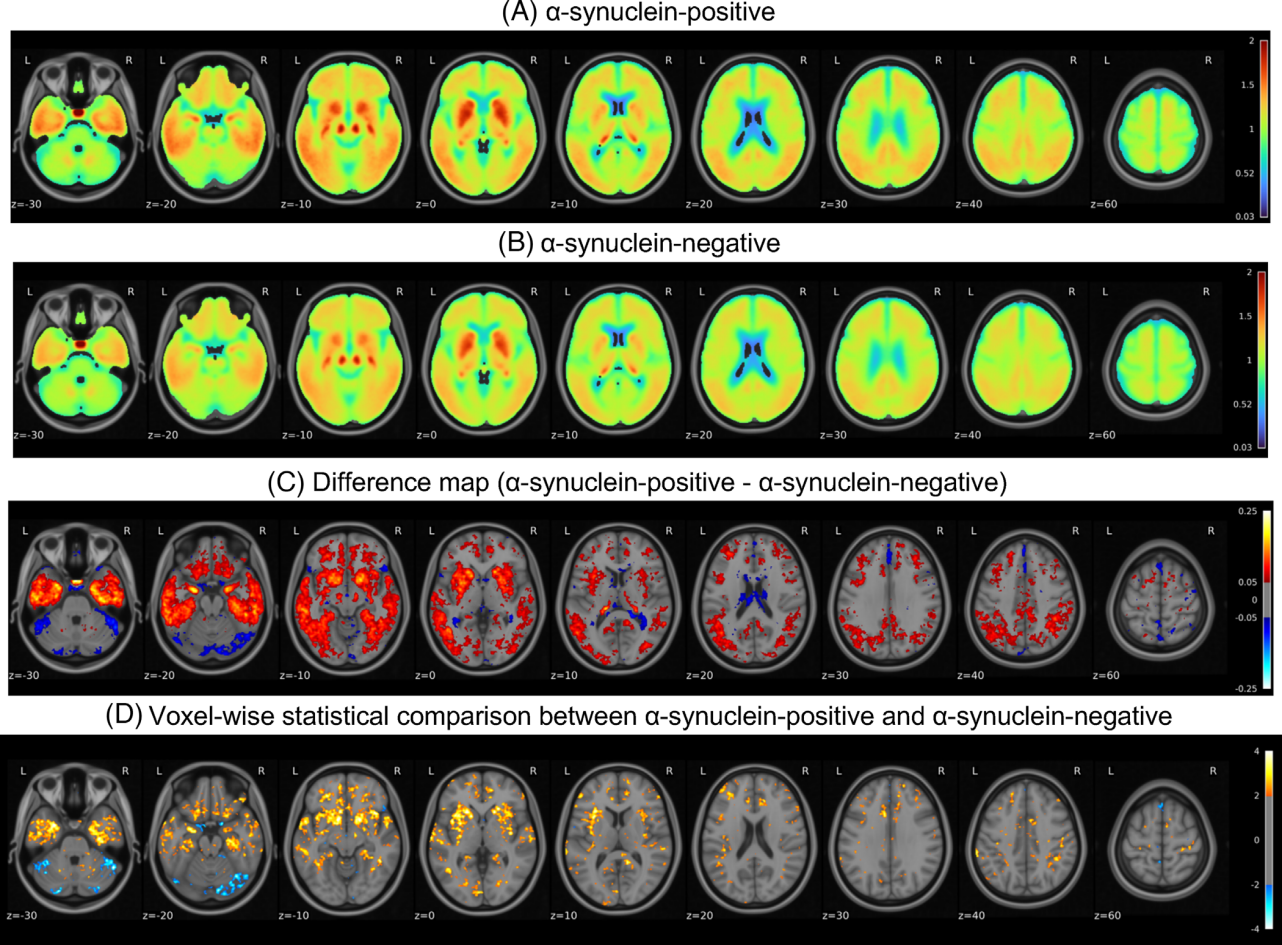
Voxel‐wise comparison of tau PET signal between α‐synuclein–positive and α‐synuclein–negative individuals (*N* = 531), controlling for age, sex, and Aβ status. A, Mean tau PET SUVR, α‐synuclein‐positive. B, Mean tau PET SUVR, α‐synuclein‐negative. C, Difference map (α‐synuclein‐positive—α‐synuclein‐negative). D, Voxel‐wise GLM implemented, shown uncorrected at *p* < 0.001 to illustrate the overall spatial pattern of group differences; these maps are not corrected for multiple comparisons. Panels (A) and (B) share an identical sequential color scale (turbo colormap, 0.03–2.0 SUVR) for direct visual comparison; panel (C), thresholded at |difference| > 0.05 to suppress near‐zero noise uses a diverging color scale, in which red and yellow indicate higher tau PET signal in α‐synuclein–positive participants and dark blue and light blue indicate higher signal in α‐synuclein–negative participants; in panel (D) the color scale indicates the *T* statistic for the group contrast (α‐synuclein positive vs. α‐synuclein negative), with red (*T* = 2) to yellow (*T* > 4) representing regions of increased tau PET signal and dark blue (*T* = −2) to light blue (*T* < −4) representing regions of decreased tau PET signal. Aβ, amyloid beta; GLM, generalized linear model; PET, positron emission tomography; SUVR, standardized uptake value ratio.

We then analyzed 531 tau PET scans using voxel‐wise analyses comparing α‐synuclein–positive and α‐synuclein–negative individuals, controlling for age, sex, and Aβ status. We also repeated the analyses including the time difference between CSF sampling and tau PET as an additional covariate. The results were almost identical, indicating that this time difference does not impact our model. As shown in Figure 1D, α‐synuclein–positive individuals showed elevated PET signal in multiple cortical and subcortical regions, particularly in the BG, insula, and temporal lobe compared to α‐synuclein–negative individuals. Decreased PET signal in α‐synuclein–positive individuals was limited to the cerebellum. After cluster‐wise FDR correction, we identified seven significant clusters (Figure 1D), listed in Table 3, corresponding to bilateral, largely symmetric regions in the BG, insula, and MTL.

**TABLE 3:** Regions of significantly greater tau deposition in α‐synuclein–positive versus α‐synuclein–negative individuals identified via voxel‐wise analysis in the full group (*N* = 531). Regions correspond to bilateral BG, insula, and MTL.

Regions	MNI coordinates (X, Y, Z)	Peak *Z* score of local maxima
Left BG	−19, 19, −2	4.67^****^
Right BG	19, 21, −3	4.94^****^
Left Insula	−30, 21, 7	4.59^**^
Right Insula	38, 19, ‐8	4.54^**^
Left MTL	−26, 2, −30	4.32^****^
Right MTL	36, −9, −34; 32, −17, −29	4.10^*^; 4.08^**^

**** : FDR *p* < 0.0001; ^***^: FDR *p* < 0.001; ^**^: FDR *p* < 0.01; ^*^: FDR *p* < 0.05.

Abbreviations: BG, basal ganglia; FDR, false discovery rate; MNI, Montreal Neurological Institute; MTL, medial temporal lobe.

As sensitivity analyses, we repeated the analyses in Aβ‐positive and Aβ‐negative groups. When the analysis was restricted to Aβ‐positive participants, results were similar except that the BG regions no longer showed significant tau differences between α‐synuclein–positive and α‐synuclein–negative individuals (Figure S1A in supporting information). In contrast, in Aβ‐negative participants, the BG was the only region with higher tau in α‐synuclein–positive individuals (Figure S1B). Cluster‐wise FDR correction did not survive across bilateral regions in the BG, insula, and MTL (Table S1 in supporting information).

### 3.3 Correlation between regional tau PET and clinical features

Because all measures significantly deviated from normality (*p* < 0.001), Spearman partial correlations were used for all statistical analyses.

#### 3.3.1 Analyses of all participants

Partial correlation analyses controlling for age, sex, and Aβ status in the full sample (*N* = 400) showed that more severe psychiatric symptoms (NPI total score) were associated with greater tau PET signal (Figure 2A) in all three bilateral regions: BG (ρ = 0.15, *p* = 0.003, FDR *p* = 0.02), insula (ρ = 0.10, *p* = 0.05, FDR *p* = 0.08), and MTL (ρ = 0.14, *p* = 0.006, FDR *p* = 0.02). Worse psychomotor functioning (PC1, reflecting slower response time across three tasks) was associated (Figure 2B) with higher BG tau PET signal (ρ = 0.11, *p* = 0.028, FDR *p* = 0.056).

**FIGURE 2 alz71932-fig-0002:**
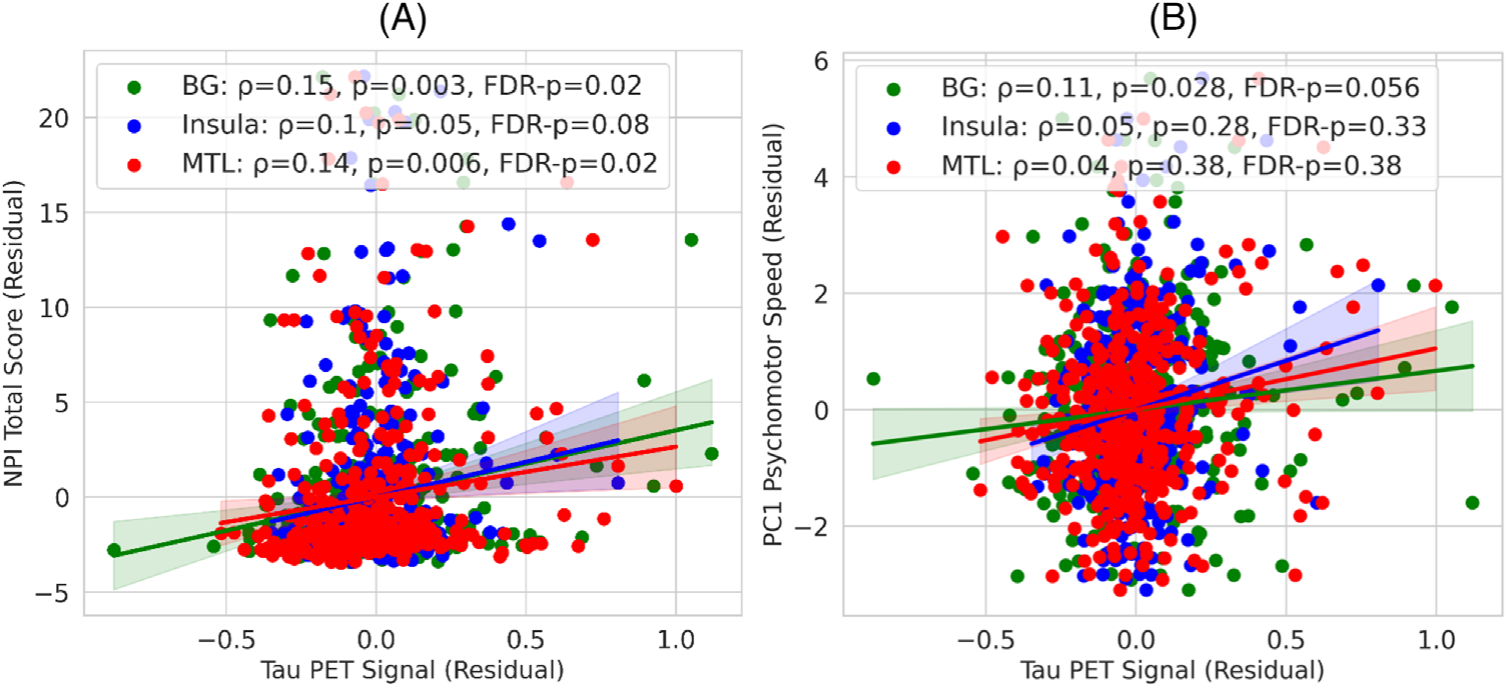
Associations between regional tau PET signal and clinical features across all participants (*N* = 400). A, Partial regression plots showing the association between psychiatric symptom severity (NPI total score) and tau PET signal in the bilateral BG, insula, and MTL. B, Partial regression plots showing the association between psychomotor speed (PC1) and tau PET signal in the same regions. Each point represents an individual participant; both axes show residuals after adjusting for age, sex, and Aβ status. Spearman ρ and FDR‐corrected *p* values are reported within each panel. Psychiatric symptom severity remained significantly associated with tau PET signal in the BG (FDR *p* = 0.02) and MTL (FDR *p* = 0.02) after correction; the insula association did not survive correction (FDR *p* = 0.08). Psychomotor function was associated with BG tau PET signal at trend level only (FDR *p* = 0.056); no association with insula or MTL tau PET signal survived correction. Aβ, amyloid beta; BG, basal ganglia; FDR, false discovery rate; MTL, medial temporal lobe; NPI, Neuropsychiatric Inventory; PC1, first principal component; PET, positron emission tomography.

#### 3.3.2 Analyses in α‐synuclein–positive group

Partial correlation analyses in the α‐synuclein–positive group (*N* = 67) showed that more severe psychiatric symptoms were associated (Figure 3A) with greater tau PET signals in two of the three regions: BG (ρ = 0.32, *p* = 0.01, FDR *p* = 0.04) and insula (ρ = 0.27, *p* = 0.03, FDR *p* = 0.05). Worse psychomotor functioning (slower response time across three tasks) was also associated with higher tau PET signal (Figure 3B) in these same two regions: BG (ρ = 0.27, *p* = 0.03, FDR *p* = 0.05) and insula (ρ = 0.30, *p* = 0.01, FDR *p* = 0.04).

**FIGURE 3:**
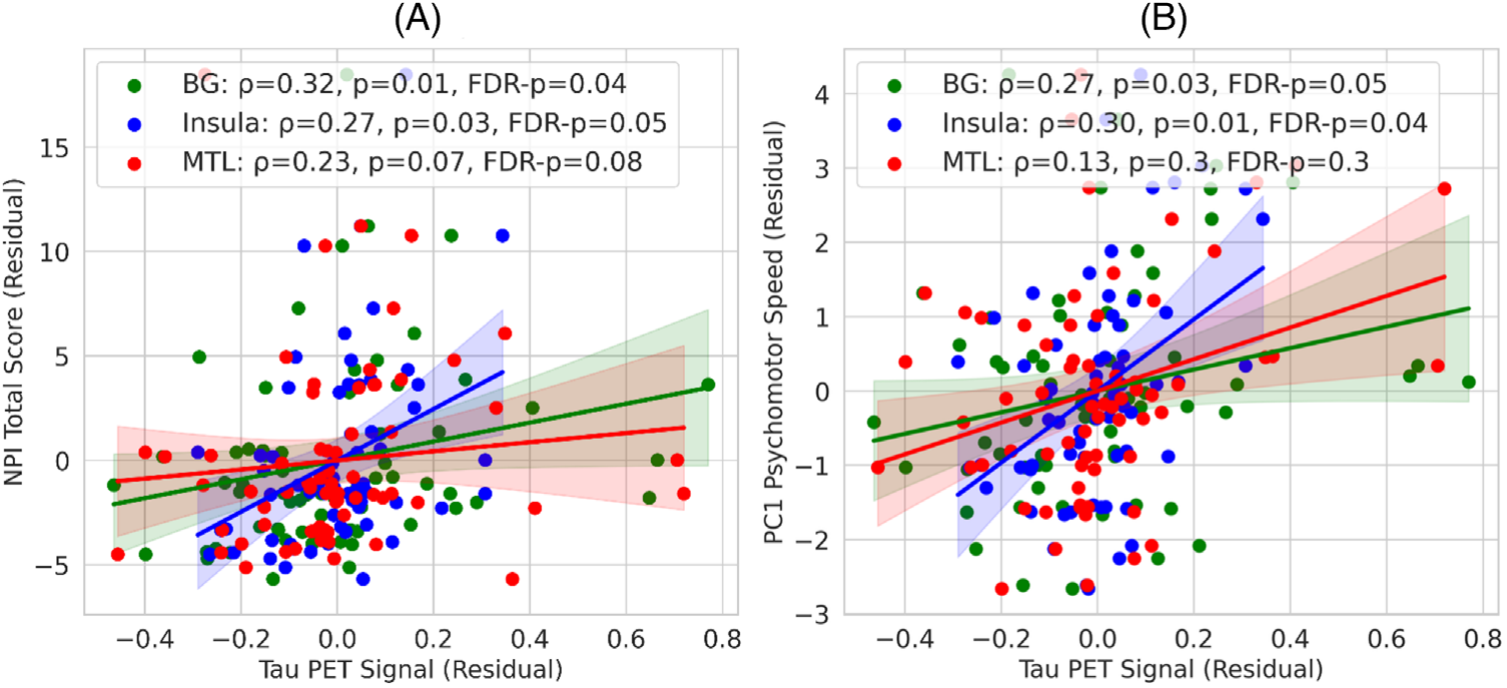
Associations between regional tau PET signal and clinical features in the α‐synuclein–positive group (*N* = 67). A, Partial regression plots showing the association between psychiatric symptom severity (NPI total score) and tau PET signal in the bilateral BG, insula, and MTL. B, Partial regression plots showing the association between psychomotor function (PC1) and tau PET signal in the same regions. Each point represents an individual participant; both axes show residuals after adjusting for age, sex, and Aβ status. Spearman ρ and FDR‐corrected *p* values are reported within each panel. Associations with BG and insula tau PET signal remained significant after correction for both psychiatric symptoms (BG: FDR *p* = 0.04; insula: FDR *p* = 0.05) and psychomotor function (BG: FDR *p* = 0.05; insula: FDR *p* = 0.04); the MTL association did not survive correction. Aβ, amyloid beta; BG, basal ganglia; FDR, false discovery rate; MTL, medial temporal lobe; NPI, Neuropsychiatric Inventory; PC1, first principal component; PET, positron emission tomography.

#### 3.3.3 Analyses in α‐synuclein–negative group

As shown in Figure S2, there were no robust correlations between clinical features and tau PET signals in any of the three regions in the α‐synuclein–negative group (*N* = 333). However, there was a marginally significant trend toward greater psychiatric symptoms associated with greater tau PET signal in the MTL (Figure S2A, ρ = 0.11, *p* = 0.04, FDR *p* = 0.22). The absence of significant associations in this group (Figure S2), five times larger than the α‐synuclein–positive group, supports the specificity of the associations reported[Fig alz71932-fig-0002] in Figure 3.

To characterize the magnitude of these associations independent of significance thresholds, each partial Spearman correlation was converted to an equivalent Cohen *d*. Across all six region–clinical features pairs, the converted effect size was consistently larger in the α‐synuclein–positive group than in the α‐synuclein–negative group: BG–psychiatric (*d* = 0.67 vs. 0.18), BG–psychomotor speed (*d* = 0.55 vs. 0.14), insula–psychiatric (*d* = 0.53 vs. 0.10), insula–psychomotor speed (*d* = 0.67 vs. 0.03), MTL–psychiatric (*d* = 0.46 vs. 0.23), and MTL–psychomotor speed (*d* = 0.26 vs. 0.07). Despite the substantially larger sample size in the α‐synuclein–negative group, every α‐synuclein–positive group achieved a larger effect size.

#### 3.3.4 Sensitivity analyses on regional tau and clinical features associations in Aβ‐positive participants

We first used the Kruskal–Wallis test to assess the overall difference in tau PET signal across the three clinical stages (HC, MCI, AD). Among Aβ‐positive participants, tau PET signal increased monotonically with clinical stage in all three ROIs: BG (HC: mean = 1.49, MCI: mean = 1.59, AD: mean = 1.80; *H* = 13.84, *p* = 0.001), insula (HC: mean = 1.08, MCI: mean = 1.16, AD: mean = 1.50; *H* = 17.81, *p* = 0.0001), and MTL (HC: mean = 1.28, MCI: mean = 1.48, AD: mean = 1.92; *H* = 33.56, *p* < 0.0001); all three comparisons survived FDR correction (*p* < 0.001). This confirms that tau PET signal tracks disease severity as expected within biologically defined AD, providing a validity check for our regional measures.

We repeated the tau–clinical correlations restricted to Aβ‐positive and α‐synuclein–positive participants (*n* = 28); the tau–clinical correlations were not only maintained but were numerically stronger than in the unrestricted α‐synuclein–positive sample. In Aβ‐positive, α‐synuclein–positive participants, tau PET signal was significantly associated with slower psychomotor speed in all three regions after FDR correction: BG (ρ = 0.45, *p* = 0.024, FDR *p* = 0.05), insula (ρ = 0.45, *p* = 0.023, FDR *p* = 0.05), and MTL (ρ = 0.48, *p* = 0.016, FDR *p* = 0.05). The psychiatric‐symptom correlations showed the same direction but did not survive correction (BG: ρ = 0.37, *p* = 0.068, FDR *p* = 0.10; insula: ρ = 0.31, *p* = 0.13, FDR *p* = 0.16; MTL: ρ = 0.20, *p* = 0.35, FDR *p* = 0.35).

## 4 DISCUSSION

In this study, we uniquely leveraged a large, well‐characterized cohort of older adults and whole‐brain voxel‐wise analyses to show that individuals with α‐synuclein pathology (based on highly accurate CSF assay) exhibit region‐specific increases in tau PET signal in the BG, insula, and MTL. These three regions, identified using unbiased, whole‐brain analysis, are key neural hubs essential for motor function,^28^, ^29^ emotional processing,^30^ and memory,^19^ respectively. While all these domains are ultimately affected when neurodegeneration progresses to severe dementia, the timing of decline across these domains differs significantly depending on the specific underlying etiology. In AD, anterograde memory impairment is typically the earliest detectable symptom,^31^, ^32^ whereas DLB is defined clinically by early motor and psychiatric dysfunction, usually with preserved memory.^1^, ^2^ In accord with these distinguishing clinical profiles, we found that across the full cohort, greater psychomotor dysfunction (estimated as a composite score derived from reaction time from three computerized tasks) was associated with greater tau PET signal in the BG, and greater severity of psychiatric symptoms, measured using the NPI, was associated with greater tau PET signal in all three regions. Notably, these relationships were significant only among α‐synuclein–positive participants, emphasizing their potential relevance for understanding the pathophysiology of synucleinopathy and the clinical heterogeneity observed across neurodegenerative diseases.

Our finding of greater tau PET signal in BG, primarily the putamen and caudate, in association with both synucleinopathy and psychomotor slowing, is novel, but is consistent with the well‐known role of BG circuitry in driving the motor features of PD and DLB. ^28^, ^29^ Notably, the spatial pattern of tau deposition we observed mirrors the dopaminergic BG structures highlighted by dopamine transporter single‐photon emission computed tomography (DAT‐SPECT), which is used clinically to support the diagnosis of PD and DLB.^33^ While these regions are not typically associated with early tau deposition in AD, and tau PET signal in BG is often attributed solely to off‐target tracer binding, ^34^ there is neuropathological evidence for tau deposition in the BG, particularly in the putamen, and nigro‐striatial tauopathy has been associated with subtle motor dysfunction in individuals with and without AD.^35^, ^36^ These findings are in accord with our demonstration that BG tau PET signal correlates with subtle motor dysfunction, and is robustly associated with the presence of α‐synuclein.

The insula also stands out as particularly distinctive pattern of tau. Although the insula is vulnerable in late‐stage AD,^37^, ^38^ here the insular tau effect was clearly modulated by α‐synuclein.^39^ We found that in α‐synuclein–positive individuals, but not α‐synuclein–negative individuals, higher insular tau was strongly associated with worse psychiatric symptoms, as well as psychomotor slowing. Our results fit with multiple neuroimaging studies implicating dysfunction of the insula with emotional dysregulation and psychiatric disease,^30^ with specific evidence for early insular dysfunction in DLB, including neuroimaging studies showing preferential insula atrophy in early DLB^39^, ^40^, ^41^ and autopsy series reporting high (phosphorylated) tau burden in the anterior insula of DLB cases with links to dementia severity.^37^, ^42^ Our in vivo findings suggest that insular tau may be a neural substrate connecting α‐synuclein pathology to emotional dysfunction. Thus, insular tau deposition might be a relatively specific biomarker of synucleinopathy and highly relevant to understanding the neural basis of disabling psychiatric symptoms in DLB. ^9^, ^43^

Stratifying by Aβ status revealed intriguing differences in patterns of tauopathy associated with the presence of α‐synuclein. In Aβ‐positive participants, α‐synuclein–positive individuals showed greater tau in the insula and especially the MTL, but not in the BG. In contrast, in Aβ‐negative participants, α‐synuclein–positive subjects exhibited higher tau confined to the BG. Results in the Aβ‐positive group are consistent with the presence of α‐synuclein enhancing tau deposition in typical AD regions like the MTL,^44^, ^45^, ^46^ with classic Braak‐like cortical tau progression.^19^, ^20^, ^32^, ^47^, ^48^, ^49^, ^50^ This is consistent with the recent finding that the presence of α‐synuclein accelerates AD pathology,^13^, ^14^ and the increasingly supported general concept that co‐pathologies appear to accelerate each other. In contrast, in the Aβ‐negative group, who by definition do not have AD, the presence of synucleinopathy was not associated with tau deposition in typical AD regions like the MTL (even with the larger sample size of the Aβ‐negative group compared to the Aβ‐positive group). Rather, in Aβ‐negative individuals, the presence of synucleinopathy was associated with greater tau PET signal limited to the BG, primarily the putamen and caudate.

Despite the high prevalence of α‐synuclein co‐pathology, clinical diagnosis remains difficult. Meta‐analytic data show that nearly 20% of DLB diagnoses based solely on clinical criteria are incorrect, and caregiver reports indicate that 78% of patients receive alternative diagnoses before DLB is correctly identified.^4^, ^51^, ^52^ The recent availability of diagnostic tests for α‐synuclein using skin biopsy and CSF assays helps address this diagnostic challenge, but gaps remain. First, skin biopsy and LP are considered invasive by many patients, and even when results of these tests are available, they are binary (positive or negative) and cannot provide information about disease stage or severity, nor the extent or anatomic location of α‐synuclein deposition in the brain. Unlike for Aβ and tau, validated in vivo PET tracers for α‐synuclein do not yet exist; first‐generation candidates are not yet suitable for clinical or broad research applications.^5^, ^6^ While not a substitute for an α‐synuclein PET tracer that could quantify neuroanatomically localized α‐synuclein deposition, our identification of a signature pattern of tau PET with corresponding clinical effects in association with α‐synuclein pathology takes an important step toward developing multimodal neuroimaging biomarkers capable of non‐invasive, early detection of synucleinopathy and contributes to improved understanding of how interactions among α‐synuclein, tau, and Aβ lead to heterogeneous clinical manifestations and clinical trajectories of neurodegeneration.

This study has several limitations that should be considered in future research. First, our sample size, particularly within the α‐synuclein–positive subgroup, was our main limitation. Despite this limited sample size, we applied FDR correction across all tested associations and used Cohen *d* to ensure our results were not driven by chance. Second, the ADNI cohort is not optimized for synucleinopathy research. ADNI does not include α‐synuclein–specific clinical batteries, motor scales tailored to PD or DLB, measures of autonomic functioning, nor comprehensive neuropsychiatric assessments sensitive to early α‐synuclein–related changes. As a result, our ability to fully characterize α‐synuclein–driven cognitive, motor, and behavioral features is limited by the available instruments. Third, use of a first‐generation tau PET tracer with significant off‐target binding complicates tau PET interpretation, especially in the medial temporal lobes. Mitigating against this, our novel results linking tauopathy to α‐synuclein status were strongest in the insula and BG. The insula is not a region with significant off‐target binding, and while off‐target binding in BG is a known issue discussed above, it does not differ between first‐ and second‐generation tau PET tracers.^53^ Larger, dedicated synucleinopathy cohorts with disease‐specific clinical measures and optimal tau PET^19^, ^49^ will be essential to validate and extend these findings.

In conclusion, we identified three brain regions (BG, insula, and MTL) where the presence of α‐synuclein pathology was associated with greater tau PET signal, and we linked these regional PET patterns to clinical features relevant to synucleinopathies. These findings help explain clinical heterogeneity across neurodegenerative presentations and emphasize the importance of considering α‐synuclein co‐pathology in clinical and translational research. In addition to improving diagnostic accuracy, recognizing these regional interactions between α‐synuclein status and tau pathology may advance understanding of how neurodegenerative pathologies, alone and in combination, produce specific symptoms, and can inform targeted interventions.

## AUTHOR CONTRIBUTIONS

S.H.H. and T.B. conceived the main conceptual ideas, performed the data analysis, and drafted the manuscript. S.H.H., T.B., and G.C.C. contributed to data interpretation, and all other authors participated in manuscript revisions and provided critical intellectual input.

## CONFLICT OF INTEREST STATEMENT

The authors declare that they have no relevant financial or non‐financial interests to disclose. This research was conducted in the absence of any commercial or financial relationships that could be construed as a potential conflict of interest. Author disclosures are available in the Supporting Information.

## ETHICS STATEMENT

As per Alzheimer's Disease Neuroimaging Initiative (ADNI) protocols, all procedures conducted in studies involving human participants adhered to ethical standards. The ADNI data collection was conducted following written informed consent from the participants. Further details can be found at adni.loni.usc.edu.

## CONSENT STATEMENT

All human subjects provided informed consent according to the ADNI study protocol, approved by the institutional review boards of all participating sites.

## Supporting information



Supporting information

Supporting information

## Data Availability

Publicly available datasets were analyzed in this study. These data can be found here: adni.loni.usc.edu.

## References

[1] Sadiq D , Whitfield T , Lee L , Stevens T , Costafreda S , Walker Z . Prodromal dementia with Lewy bodies and prodromal Alzheimer's Disease: a comparison of the cognitive and clinical profiles. J Alzheimers Dis. 2017;58(2):463‐470. doi:10.3233/JAD-161089 28453473

[2] McKeith I , Mintzer J , Aarsland D , et al. Dementia with Lewy bodies. Lancet Neurol. 2004;3:19‐28. doi:10.1016/S1474-4422(03)00619-7 14693108

[3] Galvin JE . Improving the clinical detection of Lewy body dementia with the Lewy body composite risk score. Alzheimers Dement (Amst). 2015;1(3):316‐324. doi:10.1016/j.dadm.2015.05.004 PMC4576496 26405688

[4] Rizzo G , Arcuti S , Copetti M , et al. Accuracy of clinical diagnosis of dementia with Lewy bodies: a systematic review and meta‐analysis. J Neurol Neurosurg Psychiatry. 2018;89(4):358‐366. doi:10.1136/jnnp-2017-316844 29030419

[5] Pees A , Tong J , Birudaraju S , et al. Development of pyridothiophene compounds for PET imaging of α‐Synuclein. Chem Eur J. 2024;30(23):e202303921. doi:10.1002/chem.202303921 PMC12189351 38354298

[6] Alzghool OM , van Dongen G , van de Giessen E , Schoonmade L , Beaino W . α‐Synuclein radiotracer development and in vivo imaging: recent advancements and new perspectives. Mov Disord. 2022;37(5):936‐948. doi:10.1002/mds.28984 PMC9310945 35289424

[7] Mckeith IG , Sci M , Boeve BF , et al. Diagnosis and management of dementia with Lewy bodies fourth consensus report of the DLB Consortium. Neurology. 2017;89(1):88‐100. 10.1212/WNL.0000000000004058 PMC5496518 28592453

[8] Rossi M , Candelise N , Baiardi S , et al. Ultrasensitive RT‐QuIC assay with high sensitivity and specificity for Lewy body‐associated synucleinopathies. Acta Neuropathol. 2020;140(1):49‐62. doi:10.1007/s00401-020-02160-8 PMC7299922 32342188

[9] Butler T . Skin biopsy diagnosis of late‐onset psychosis as prodromal dementia with Lewy bodies: a case series. J Neuropsychiatry Clin Neurosci. 2026;38(3):278‐282. doi:10.1176/appi.neuropsych.20250066 41837743

[10] Gibbons CH , Levine T , Adler C , et al. Skin biopsy detection of phosphorylated α‐synuclein in patients with synucleinopathies. JAMA, J Am Med Assoc. 2024;331(15):1298‐1306. doi:10.1001/jama.2024.0792 PMC10955354 38506839

[11] Tosun D , Hausle Z , Iwaki H , et al. A cross‐sectional study of α‐synuclein seed amplification assay in Alzheimer's disease neuroimaging initiative: prevalence and associations with Alzheimer's disease biomarkers and cognitive function. Alzheimers Dement. 2024;20(8):5114‐5131. doi:10.1002/alz.13858 PMC11350010 38770829

[12] Bolsewig K , Bellomo G , Hok‐A‐Hin YS , et al. A quantitative DOPA decarboxylase biomarker for diagnosis in Lewy body disorders. Nat Med. 2026;32(3):1073‐1084. doi:10.1038/s41591-026-04212-0 PMC13004688 41699128

[13] Pichet Binette A , Mammana A , Wisse L , et al. Associations between misfolded alpha‐synuclein aggregates and Alzheimer's disease pathology in vivo. Alzheimers Dement. 2024;20(11):7624‐7634. doi:10.1002/alz.14225 PMC11567871 39258841

[14] Franzmeier N , Roemer‐Cassiano SN , Bernhardt AM , et al. Alpha synuclein co‐pathology is associated with accelerated amyloid‐driven tau accumulation in Alzheimer's disease. Mol Neurodegener. 2025;20(1):31. doi:10.1186/s13024-025-00822-3 PMC11916967 40098057

[15] Abu‐Rumeileh S , Arajyan G , Reiman EM , Otto M , Weise CM . Contribution of alpha‐synuclein pathology to cerebral glucose metabolism in patients with amnestic MCI. Alzheimers Dement. 2024;20(10):7411‐7419. doi:10.1002/alz.14151 PMC11485405 39177111

[16] Mak E , Przybelski SA , Wiste HJ , et al. Influence of alpha‐synuclein on glucose metabolism in Alzheimer's disease continuum: analyses of α‐synuclein seed amplification assay and FDG‐PET. Alzheimers Dement. 2025;21(2):e14571. doi:10.1002/alz.14571 PMC11815207 39935389

[17] Williams T , Sorrentino Z , Weinrich M , Giasson BI , Chakrabarty P . Differential cross‐seeding properties of tau and α‐synuclein in mouse models of tauopathy and synucleinopathy. Brain Commun. 2020;2(2):fcaa090. doi:10.1093/braincomms/fcaa090 PMC7567170 33094280

[18] Bassil F , Meymand ES , Brown HJ , et al. α‐Synuclein modulates tau spreading in mouse brains. J Exp Med. 2021;218(1):e20192193. doi:10.1084/JEM.20192193 PMC7588140 33091110

[19] Hojjati SH , Chiang GC , Butler TA , et al. Heterogeneous tau deposition patterns in the preclinical stage link to domain‐specific cognitive deficits. Alzheimers Dement. 2025;21(5):e70153. doi:10.1002/alz.70153 PMC12069010 40355988

[20] Jagust W . Imaging the evolution and pathophysiology of Alzheimer disease. Nat Rev Neurosci. 2018;19(11):687‐700. doi:10.1038/s41583-018-0067-3 PMC7032048 30266970

[21] Maruff P , Thomas E , Cysique L , et al. Validity of the CogState brief battery: relationship to standardized tests and sensitivity to cognitive impairment in mild traumatic brain injury, schizophrenia, and AIDS dementia complex. Arch Clin Neuropsychol. 2009;24(2):165‐178. doi:10.1093/arclin/acp010 19395350

[22] Mielke MM , Machulda MM , Hagen CE , et al. Performance of the CogState computerized battery in the Mayo Clinic Study on Aging. Alzheimer's and Dementia. 2015;11(11):1367‐1376. doi:10.1016/j.jalz.2015.01.008 PMC4595161 25858683

[23] Evarts EV , Teräväinen H , Calne DB . Reaction time in Parkinson's disease. Brain. 1981;104:167‐186. doi:10.1093/brain/104.1.167 7470841

[24] Greve DN , Svarer C , Fisher PM , et al. Cortical surface‐based analysis reduces bias and variance in kinetic modeling of brain PET data. Neuroimage. 2014;92:225‐236. doi:10.1016/j.neuroimage.2013.12.021 PMC4008670 24361666

[25] Smith SM , Jenkinson M , Woolrich MW , et al. Advances in functional and structural MR image analysis and implementation as FSL. Neuroimage. 2004;23:S208‐S219. doi:10.1016/j.neuroimage.2004.07.051 15501092

[26] Avants B , Tustison N , Johnson H . Advanced Normalization Tools (ANTS). Insight Journal. 2009;2:1‐35.

[27] Ashburner J . Computational anatomy with the SPM software. Magn Reson Imaging. 2009;27(8):1163‐1174. doi:10.1016/j.mri.2009.01.006 19249168

[28] Helie S , Chakravarthy S , Moustafa AA . Exploring the cognitive and motor functions of the basal ganglia: an integrative review of computational cognitive neuroscience models. Front Comput Neurosci. 2013;7:174. doi:10.3389/fncom.2013.00174 PMC3854553 24367325

[29] Caligiore D , Pezzulo G , Baldassarre G , et al. Consensus paper: towards a systems‐level view of cerebellar function: the interplay between cerebellum, basal ganglia, and cortex. Cerebellum. 2017;16(1):203‐229. doi:10.1007/s12311-016-0763-3 PMC5243918 26873754

[30] Kelly C , Toro R , Di Martino A , et al. A convergent functional architecture of the insula emerges across imaging modalities. Neuroimage. 2012;61(4):1129‐1142. doi:10.1016/j.neuroimage.2012.03.021 PMC3376229 22440648

[31] Hojjati SH , Babajani‐Feremi A . Seeing beyond the symptoms: biomarkers and brain regions linked to cognitive decline in Alzheimer's disease. Front Aging Neurosci. 2024;16:1356656. 10.3389/fnagi.2024.1356656 PMC11135344 38813532

[32] Hojjati SH , Feiz F , Ozoria S , Razlighi QR . Topographical overlapping of the amyloid‐β and tau pathologies in the default mode network predicts Alzheimer's disease with higher specificity. J Alzheimers Dis. 2021;83(1):407‐421. doi:10.3233/JAD-210419 34219729

[33] O'Shea DM , Arkhipenko A , Galasko D , et al. Practical use of DAT SPECT imaging in diagnosing dementia with Lewy bodies: a US perspective of current guidelines and future directions. Front Neurol. 2024;15:1395413. doi:10.3389/fneur.2024.1395413 PMC11073567 38711561

[34] Lyoo CH , Cho H , Choi JY , Ryu YH , Lee MS . Tau positron emission tomography imaging in degenerative Parkinsonisms. J Mov Disord. 2018;11(1):1‐12. doi:10.14802/jmd.17071 PMC5790630 29381890

[35] Chu Y , Hirst WD , Federoff HJ , Harms AS , Stoessl AJ , Kordower JH . Nigrostriatal tau pathology in parkinsonism and Parkinson's disease. Brain. 2024;147(2):444‐457. doi:10.1093/brain/awad388 PMC10834249 38006313

[36] Hamasaki H , Honda H , Suzuki SO , et al. Tauopathy in basal ganglia involvement is exacerbated in a subset of patients with Alzheimer's disease: the Hisayama study. Alzheimers Dement (Amst). 2019;11:415‐423. doi:10.1016/j.dadm.2019.04.008 PMC6558096 31206007

[37] Bonthius DJ , Solodkin A , Van Hoesen GW . Pathology of the insular cortex in Alzheimer disease depends on cortical architecture. J Neuropathol Exp Neurol. 2005;64(10):910‐922. doi:10.1097/01.jnen.0000182983.87106.d1 16215463

[38] Mattsson N , Palmqvist S , Stomrud E , Vogel J , Hansson O . Staging β ‐Amyloid Pathology with Amyloid Positron Emission Tomography. JAMA Neurol. 2019;76(11):1319‐1329. doi:10.1001/jamaneurol.2019.2214 PMC6646987 31314895

[39] Blanc F , Colloby SJ , Philippi N , et al. Cortical thickness in dementia with lewy bodies and alzheimer's disease: a comparison of prodromal and dementia stages. PLoS One. 2015;10(6):e0127396. doi:10.1371/journal.pone.0127396 PMC4489516 26061655

[40] Roquet D , Noblet V , Anthony P , et al. Insular atrophy at the prodromal stage of dementia with Lewy bodies: a VBM DARTEL study. Sci Rep. 2017;7(1):9437. doi:10.1038/s41598-017-08667-7 PMC5573371 28842567

[41] Christopher L , Koshimori Y , Lang AE , Criaud M , Strafella AP . Uncovering the role of the insula in non‐motor symptoms of Parkinson's disease. Brain. 2014;137(8):2143‐2154. doi:10.1093/brain/awu084 PMC4107733 24736308

[42] Fathy YY , Jonkman LE , Bol JJ , et al. Axonal degeneration in the anterior insular cortex is associated with Alzheimer's co‐pathology in Parkinson's disease and dementia with Lewy bodies. Transl Neurodegener. 2022;11(1):52. doi:10.1186/s40035-022-00325-x PMC9728006 36474289

[43] Hojjati SH , Chen K , Chiang GC , et al. Utilizing structural MRI and unsupervised clustering to differentiate schizophrenia and Alzheimer's disease in late‐onset psychosis. Behav Brain Res. 2025;480:115386. doi:10.1016/j.bbr.2024.115386 PMC12040479 39644998

[44] Hojjati SH , Chiang GC , Butler TA , et al. Remote associations between tau and cortical amyloid‐β are stage‐dependent. J Alzheimers Dis. 2024;100(4):1467‐1482. doi:10.3233/JAD-231362. Preprint posted online. PMC11091581 38552116

[45] Hojjati SH , Butler TA , Chiang GC , et al. Distinct and joint effects of low and high levels of Aβ and tau deposition on cortical thickness. Neuroimage Clin. 2023;38:103409. doi:10.1016/j.nicl.2023.103409 PMC10165160 37104927

[46] Ahmadi B , Armstrong M , Asken BM , Reisi‐Gahrooei M , Babajani‐Feremi A . Deep learning‐based MRI analysis reveals Lewy body co‐pathology accelerates brain aging in Alzheimer's disease. Alzheimers Dement. 2026;22(7):e71593. doi:10.1002/alz.71593 PMC13370797 42454584

[47] Braak H , Braak E . Neuropathological stageing of Alzheimer‐related changes. Acta Neuropathol. 1991;82(4):239‐259. doi:10.1007/BF00308809 1759558

[48] Braak H , Braak E , Bohl J . Staging of Alzheimer‐related cortical destruction. Eur Neurol. 1993;33(6):403‐408. doi:10.1159/000116984 8307060

[49] Hojjati SH , Butler TA , de Leon M , et al. Inter‐network functional connectivity increases by beta‐amyloid and may facilitate the early stage of tau accumulation. Neurobiol Aging. 2025;148:16‐26. doi:10.1016/j.neurobiolaging.2025.01.005 39879839

[50] Hojjati SH , Feiz F , Nayak S , et al. Hub brain regions for the remote spatial association between tau and Amyloid‐β proteins. Alzheimer's & Dementia. 2023;19(S16):e076456. doi:10.1002/alz.076456

[51] Thomas AJ , Taylor JP , McKeith I , et al. Development of assessment toolkits for improving the diagnosis of the Lewy body dementias: feasibility study within the DIAMOND Lewy study. Int J Geriatr Psychiatry. 2017;32(12):1280‐1304. doi:10.1002/gps.4609 PMC5724510 27928840

[52] Galvin JE , Duda JE , Kaufer DI , Lippa CF , Taylor A , Zarit SH . Lewy body dementia: the caregiver experience of clinical care. Parkinsonism Relat Disord. 2010;16(6):388‐392. doi:10.1016/j.parkreldis.2010.03.007 PMC2916037 20434939

[53] Li J , Kumar A , Långström B , Nordberg A , Ågren H . Insight into the binding of first‐ and second‐generation PET tracers to 4R and 3R/4R tau protofibrils. ACS Chem Neurosci. 2023;14(18):3528‐3539. doi:10.1021/acschemneuro.3c00437 PMC10515481 37639522

