# Supplementary material for: α‐synuclein pathology drives region‐specific tau deposition
Source: Alzheimers Dement. 2026 Oct 25;22(10):e71932. doi: 10.1002/alz.71932 (PMC13696758; doi:10.1002/alz.71932)
Supplement: Supplementary file 2 [file ALZ-22-e71932-s001.docx]

**Supplementary Materials:**

(a) Aβ-positive

(b) Aβ-negative

**Figure S1**. Voxel-wise comparison of tau-PET signal between α-synuclein-positive and α-synuclein-negative individuals, stratified by Aβ status, controlling for age and sex. (a) Aβ-positive participants: results were similar to the full-sample comparison except that the basal ganglia (BG) no longer showed a significant difference between α-synuclein-positive and α-synuclein-negative individuals. (b) Aβ-negative participants: the BG was the only region showing significantly higher tau-PET signal in α-synuclein-positive individuals. Both panels are thresholded and cluster-corrected identically to the full-sample analysis (FDR < 0.001). The color scale indicates the T-statistic for the group contrast, with red/yellow representing increased tau-PET signal and dark blue/light blue representing decreased tau-PET signal in α-synuclein-positive individuals, matching the convention used in the main voxel-wise comparison.

**Table S1.** Regions of significantly greater tau deposition in α-Synuclein-positive versus α-Synuclein-negative individuals identified via voxel-wise analysis in the full group (N = 531). Regions correspond to bilateral Basal Ganglia (BG), insula, and Medial Temporal Lobe (MTL). Results in Aβ-positive (N = 236) and Aβ-negative (N = 295) subgroups are also presented.

|  | MNI Coordinates (X, Y, Z) and peak Z score of local maxima | |
| --- | --- | --- |
|  | Aβ-positive | Aβ-negative |
| Left BG | - | MNI: -20, 19, -1  Z = 3.41 |
| Right BG | - | MNI:19, 21, -3  Z = 3.81 |
| Left Insula | MNI:-31, 20, 6; -35,1,12  Z = 3.91; 3.79 | - |
| Right Insula | - | - |
| Left MTL | MNI: -30,0, -30; -33, -2, -37  Z = 3.69; 3.60 | - |
| Right MTL | MNI: 26, -19,-21; 34, -16,-30  Z = 4.04; 3.61 | - |

Aβ: Amyloid-βeta

(a)	(b)

**Figure S2.** Associations between regional tau-PET signal and clinical features in the α-synuclein-negative group (N = 333), presented as a specificity contrast to Figure 3. (a) Partial regression plots showing the association between psychiatric symptom severity (NPI total score) and tau-PET signal in the bilateral BG, insula, and MTL. (b) Partial regression plots showing the association between psychomotor function (PC1) and tau-PET signal in the same regions. Each point represents an individual participant; both axes show residuals after adjusting for age, sex, and Aβ status. No association survived FDR correction (Benjamini–Hochberg, six tests); the strongest nominal association (MTL–psychiatric symptoms, ρ = 0.11, p = 0.04) did not survive correction (FDR-p = 0.22).
